# Oxidative stress contributes to hypermethylation of Histone H3 lysine 9 in placental trophoblasts from preeclamptic pregnancies

**DOI:** 10.3389/fendo.2024.1371220

**Published:** 2024-04-25

**Authors:** Yang Gu, Danielle Cooper, David F. Lewis, Dani Zoorob, Yuping Wang

**Affiliations:** Department of Obstetrics and Gynecology, Louisiana State University Health Sciences Center, Shreveport, LA, United States

**Keywords:** placental trophoblast, H3K9 methylation, superoxide dismutase, hypoxia, oxidative stress, preeclampsia

## Abstract

**Background and objective:**

Aberrant epigenetic regulation and increased oxidative stress in the placenta play a significant role in placental pathophysiology and fetal programming in preeclampsia, a hypertensive disorder in human pregnancy. The purpose of the study is to investigate if hypermethylation of histone H3K9 occurs in placental trophoblasts from preeclampsia.

**Methods:**

Trophoblasts were isolated and cultured from 14 placentas, 7 from normotensive pregnant women and 7 from preeclamptic pregnancies. Methylated H3K9 expression and antioxidant superoxide dismutase expression were determined by Western blot. We also examined consequences of oxidative stress and the downstream effects of histone methyltransferase inhibition on H3K9 expression associated with antioxidant CuZn-SOD and Mn-SOD expression in placental trophoblasts.

**Results:**

We found that expression of mono-, di-, and tri-methylation of histone H3 lysine 9 (H3K9me1, H3K9me2 and H3K9me3) was significantly increased, p<0.01, which correlated with downregulation of antioxidant superoxide dismutase CuZn-SOD and Mn-SOD expression, in trophoblasts from preeclamptic placentas compared to those from uncomplicated control placentas. We further demonstrated hypoxia could promote histone H3K9 methylation in placental trophoblasts, and hypoxia-induced upregulation of H3K9me1, H3K9me2 and H3K9me3 expression was reversible when hypoxic condition was removed. In addition, we also uncovered that inhibition of methyltransferase not only prevented hypoxia-induced upregulation of H3K9me1, H3K9me2 and H3K9me3 expression, but also abolished hypoxia-induced downregulation of CuZn-SOD and Mn-SOD expression in placental trophoblasts.

**Conclusions:**

These findings are noteworthy and provide further evidence that increased oxidative stress in the intrauterine environment is likely a mechanism to induce aberrant histone modification in placental trophoblasts in preeclampsia. Moreover, CuZn-SOD and Mn-SOD expression/activity are possibly H3K9 methylation-dependent in placental trophoblasts, which further suggest that oxidative stress and aberrant histone modification have significant impact on placental trophoblasts/fetal programming in preeclampsia.

## Introduction

Preeclampsia, characterized by increased blood pressure and presence of proteinuria after 20 weeks of gestation, is a leading cause of both maternal and fetal morbidity and mortality worldwide. Although the cause of preeclampsia remains elusive, it is believed that the consequences of placental trophoblast dysfunction, such as increased oxidative stress, increased inflammatory response, increased microparticle deposition, and aberrant angiogenic and antiangiogenic factor production, etc. significantly impact maternal vascular function and contribute to preeclampsia development (1–3).

Rising evidence suggests that cellular function is largely regulated by the epigenetic machineries (4), which include DNA methylation, histones modification, and expression and production of miRNAs and non-coding RNAs, etc. The central importance of these epigenetic machineries is that they are conserved across all or most cellular life and can regulate gene expression at both the transcriptional and posttranscriptional levels without altering DNA sequence. Recently, there has been a rapid expansion of research in the field of epigenetics in the maternal and fetal biology. Epigenetic alterations, such as changes in DNA methylation status, altered histone modification, and aberrant miRNA expression, were reported in placental trophoblasts and fetal vessel endothelial cells in preeclampsia (5–10).

Being part of the primary interface between the mother and fetus in pregnancy, placental trophoblasts are susceptible to various bioactive or micro-environmental agents that come from maternal circulation or are produced by placental cells, which have the capacity to alter placental cell function and influence on fetal development. It is well known that an imbalance of intercellular and intracellular environments can produce alterations of gene-expression regulatory elements. Increased oxidative stress/increased production of reactive oxygen species (ROS) is one of the key pathophysiological characteristics in preeclamptic placentas (11). To determine if increased oxidative stress is associated or could induce histone modification in placental trophoblasts in preeclampsia, we specifically examined if hypermethylation of histone H3 lysine 9 (H3K9) occurs in placental trophoblasts in preeclampsia. We also examined consequences of oxidative stress on methylated H3K9 expression and the downstream effects of histone methyltransferase inhibition on H3K9 expression associated with antioxidant CuZn-SOD and Mn-SOD expression in placental trophoblasts.

## Materials and methods

### Chemicals and reagents

Dulbecco’s modified Eagle’s medium (DMEM, D5523), Percoll (P1644), antibiotic antimycotic solution (A5955), protease inhibitors, and antibody against β‐actin (A2066) were purchased from Sigma-Aldrich Chemicals (St. Louis, MO); Trypsin (LS003703) and deoxyribonuclease (LS002139) were from Worthington Biochemical Corporation (Lakewood, NJ); Fetal bovine serum (FBS) was from Atlantic Biologicals (Flowery Branch, GA); HTR-8/SVneo trophoblasts (CRL-3271) was purchased from American Type Culture Collection (ATCC) (Manassas, VA); Bicinchonininc acid (BCA) reagent was from Bio-Rad Laboratories (Hercules, CA); Antibodies against mono-methyl-histone H3 (Lys9) (D85B4, 4658), di-methyl-histone H (Lys9) (D85B4, 4658), tri-methyl-histone H3 (Lys9) (D4W1U, 13969), CuZn-SOD (SOD1, E4G1H, 37385S), and Mn-SOD (SOD2, D3X8F, 13141S) were obtained from Cell Signaling (Danvers, MA); BIX01294 (S8006) was purchased from Selleck Chemicals LLC (Houston, TX); Chemiluminescent (ECL) detection Kit was from Amersham Corp (Arlington Heights, Illinois); All other chemicals were from Sigma Chemicals unless otherwise noted.

### Placental tissue collection

Placentas from normotensive pregnant women (n = 7) and placentas from preeclamptic pregnancies (n = 7) were collected immediately after delivery from Ochsner Louisiana State University (LSU) Health hospital in Shreveport, Louisiana. Tissue collection was approved by Institutional Review Board (IRB) at LSU Health Sciences Center – Shreveport (LSUHSC-S). Normotensive pregnancy is defined as pregnancy with maternal blood pressure <140/90mmHg, and absence of proteinuria and medical and obstetrical complications. Diagnosis of preeclampsia is based on the American College Obstetricians and Gynecologists (ACOG) criteria (12): newly onset of hypertension with blood pressure ≥140mmHg systolic or ≥90mmHg diastolic with or without proteinuria after 20 weeks of gestation or new onset of hypertension plus significant end-organ dysfunction after 20 weeks of gestation or postpartum, including thrombocytopenia, renal insufficiency, impaired liver function, and pulmonary edema, or uteroplacental dysfunction, such as fetal growth retardation or abnormal Doppler ultrasound findings of uteroplacental blood flow, etc. None of the patients had signs of infection. Freshly obtained placental tissues were used for trophoblast isolation. Demographic data for normal and preeclamptic pregnant subjects is presented in **Table 1**. There was no difference in maternal age, racial status, body mass index (BMI), delivery mode, and newborn gender between normal pregnancy and preeclampsia groups. However, Maternal blood pressure was significantly higher in the preeclamptic group than in the control group. In contrast, gestational age at delivery, placental weight, and newborn weight were significantly less in the preeclamptic group than in the control group. In the preeclampsia group, 4 out of the 7 patients were early-onset cases, who were delivered <34 weeks of gestation. and 6 out of the 7 patients were delivered by caesarean section due to non-reassuring fetal heart rate tracing (NRFHT), *HELLP* (Hemolysis, Elevated Liver enzymes and Low Platelets) syndrome, or previous caesarean section.

**Table 1:** Demographic data of study subjects from whom the placenta was used in the study.

Characteristic		Normal Pregnancy n=7	Preeclampsia n=7	p value
Maternal Age		25 ± 6	30 ± 5	0.0857
Racial Status:	Black	6	7	ND
	White	0	0	ND
	Other	1	0	ND
BMI		38 ± 10	32 ± 9	0.2598
Nulliparous		2	0	0.4615
Blood Pressure (mmHg):				
	Systolic	123 ± 13	170 ± 15	<0.001
	Diastolic	67 ± 9	94 ± 12	<0.001
Gestational Age (weeks^+days^)				
at delivery		38^+6^ ± 1^+4^	32^+1^ ± 4^+6^	<0.01
Delivery Mode: VD/CS		4/3	1/6	0.5594
Newborn Gender (Male/Female)		3/4	1/6	0.5594
Newborn Weight (gram)		3,299 ± 482	1,701 ± 1,140	<0.01
Placental Weight (gram)		619 ± 131	402 ± 137	<0.05

Data are expressed as mean ± SD. ND, not determined.

BMI, Body mass index; VD, vaginal delivery; CS, cesarean section.

### Trophoblast isolation and culture

Trophoblasts from normal and preeclamptic placentas were isolated by trypsin digestion and purified by Percoll gradient centrifugation as previously described (13). Freshly isolated trophoblasts were seeded into 6-well plates (5×10^6^cells/well) and cultured under standard culturing conditions (37°C, 5% CO_2_, humidified atmosphere) in DMEM containing 10%FBS and antibiotics for 3 days. Total cellular protein was then extracted using protein lysis buffer containing protease inhibitors, collected, and stored at -80C until protein expression analysis by Western blot.

To test if H3K9 methylation in trophoblasts can be induced by oxidative stress, an *in vitro* hypoxia and re-oxygenation model was employed in the study, in which HTR-8/SVneo trophoblasts were used. HTR-8/SVneo were seeded in 6-well cluster cell culture plates (5×10^6^cells/well) and incubated with DMEM containing 5%FBS and antibiotics. After the cells reach confluence, hypoxia and re-oxygenation experiments were performed, in which HTR-8/SVneo trophoblasts were cultured in 3 settings: 1) normoxic culture: cells were cultured in a regular cell culture incubator (5%CO2/95% air = 21%O2); 2) hypoxic culture: cells were culture in a sealed portable air chamber (Billups-Rothernberg, Del Mar, CA) for 24 hours, which was flushed with 2%O2 (2%O2/5%CO2 balanced with N2); 3) hypoxia + re-oxygenation culture: cells were first cultured in a sealed portable air chamber (flushed with 2%O2), after 24 hours of hypoxic culture as the setting 2, the cell culture plates were then transferred to a regular cell culture incubator (21%O2) (re-oxygenation) for 6 hours. At the end of experiment, total cellular protein was extracted using protein lysis buffer, collected, and stored at -80C until protein expression analysis by Western blot.

### Protein expression by Western blot

Trophoblast total cellular protein was extracted at the end of cell culture experiments using protein lysis buffer (protease inhibitor cocktail, which contains phenylmethylsulfonyl fluoride (PMSF, serine protease inhibitor), dithiothreitol (DTT, reducing disulfide bonds of proteins), NaF (phosphoseryl and phosphothreonyl phosphatase inhibitor), Na3VO4 (phosphatase inhibitor), aprotinin (trypsin inhibitor), and leupeptin (cysteine, serine, and threonine inhibitor). Protein concentration in each sample was measured by using bicinchonininc acid (BCA) reagent (Bio-Rad Laboratories, Hercules, CA).

Trophoblast protein expression for H3K9me1, H3K9me2, H3K9me3, CuZn-SOD, and Mn-SOD were determined by Western blot. A standard Western blot procedure was used as described previously (14, 15). An aliquot of 10μg of total trophoblast cellular protein per sample was subject to electrophoresis (Bio-Red, Hercules, CA) and then transferred to nitrocellulose membranes. After blocking, membranes were probed with primary antibody and followed by a secondary antibody, respectively. The bound antibody was visualized with an enhanced ECL detection Kit. β-actin expression was determined and served as loading control for each sample. The band densities were scanned and analyzed by NIH Image J software. The density of β-actin expression was used to normalize H3K9me1, H3K9me2, H3K9me3, CuZn-SOD, and Mn-SOD expression for each sample.

### Data analysis

Demographic data were expressed as mean ± SD and protein expression data were presented as mean ± SE. Statistical analysis was performed with unpaired t-test, paired t-test or ANOVA using Prism computer software (GraphPad Software, Inc. La Jolla, CA). Student-Newman-Keuls test was used as post-hoc test. A probability less than 0.05 was considered as statistically significant.

## Results

### Upregulation of H3K9me1, H3K9me2, and H3K9me3 expression in placental trophoblasts from preeclamptic pregnancies

**Figure 1:**
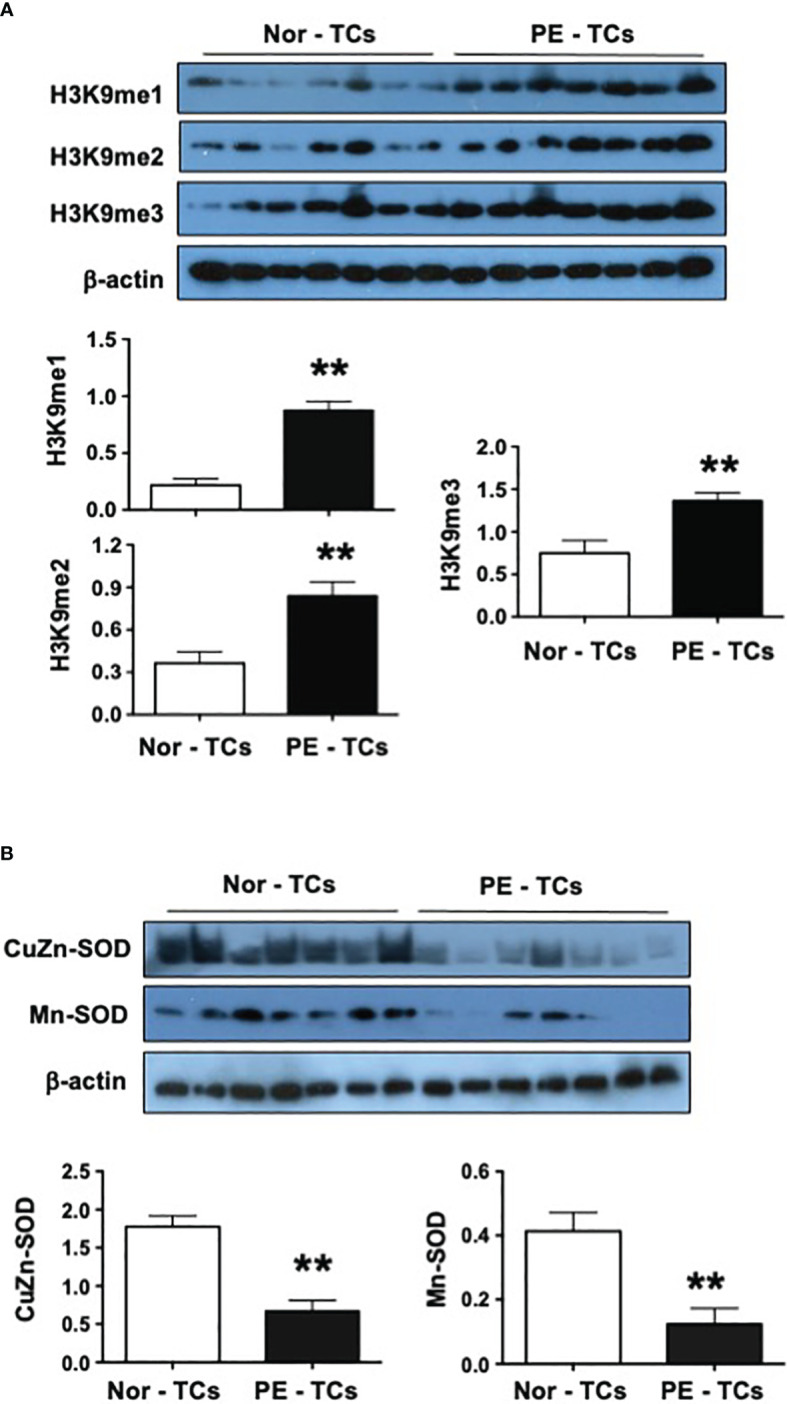
Expression of H3K9m1, H3K9m2, H3K9m2, CuZn-SOD, and Mn-SOD in placental trophoblasts from normal and preeclamptic pregnancies. **(A)** Expression of H3K9me1, H3K9me2, H3K9me3 in trophoblasts (TCs) from 7 normal (Nor) and 7 preeclamptic (PE) placentas. The bar graphs show relative expression for H3K9me1, H3K9me2, and H3K9me3 after normalized by β-actin expression in each sample. **(B)** Expression of CuZn-SOD and Mn-SOD in trophoblasts from 7 normal and 7 preeclamptic placentas. The bar graphs show relative expression for CuZn-SOD and Mn-SOD after normalized by β-actin expression in each sample. These data revealed that downregulation of antioxidant CuZn-SOD and Mn-SOD expression are associated with upregulation of methylated H3K9 expression in trophoblasts from preeclamptic vs. normal placentas. **p<0.01: PE-TCs vs. Nor-TCs.

We first determined methylated H3K9 expression in placental trophoblasts from 7 normal and 7 preeclamptic pregnancies. Three methylated forms of H3K9 (H3K9me1, H3K9me2, and H3K9me3) were examined. As shown in **Figure 1A**, expression of H3K9me1, H3K9me2, and H3K9me3 were all upregulated in trophoblasts from preeclamptic placentas compared to those from uncomplicated pregnant controls. The bar graphs show the relative H3K9me1, H3K9me2, and H3K9me3 expression after normalized with β-actin expression in each sample, p < 0.01, respectively. These data indicate the presence of hypermethylation of H3K9 in placental trophoblasts from preeclamptic compared to those from normal pregnancies.

### Downregulation of CuZn-SOD and Mn-SOD expression in placental trophoblasts from preeclamptic pregnancies

To determine if increased oxidative stress is associated with hypermethylation of H3K9 expression in preeclamptic trophoblasts, expression of two antioxidant enzymes CuZn-SOD and Mn-SOD were examined. As shown in **Figure 1B**, both CuZn-SOD and Mn-SOD expression were significantly reduced in trophoblasts from preeclamptic vs. control placenta (p<0.01). CuZn-SOD and Mn-SOD are important scavenger of superoxide anions in biological cells. CuZn-SOD locates in cytosol and Mn-SOD locates in mitochondria. Reduced CuZn-SOD and Mn-SOD expression are indicators of increased oxidative stress. These results suggest that increased oxidative stress is associated with hypermethylation status of histone H3K9, or vice versa, in placental trophoblasts in preeclampsia.

### Hypoxia promotes H3K9 methylation in placental trophoblasts

**Figure 2:**
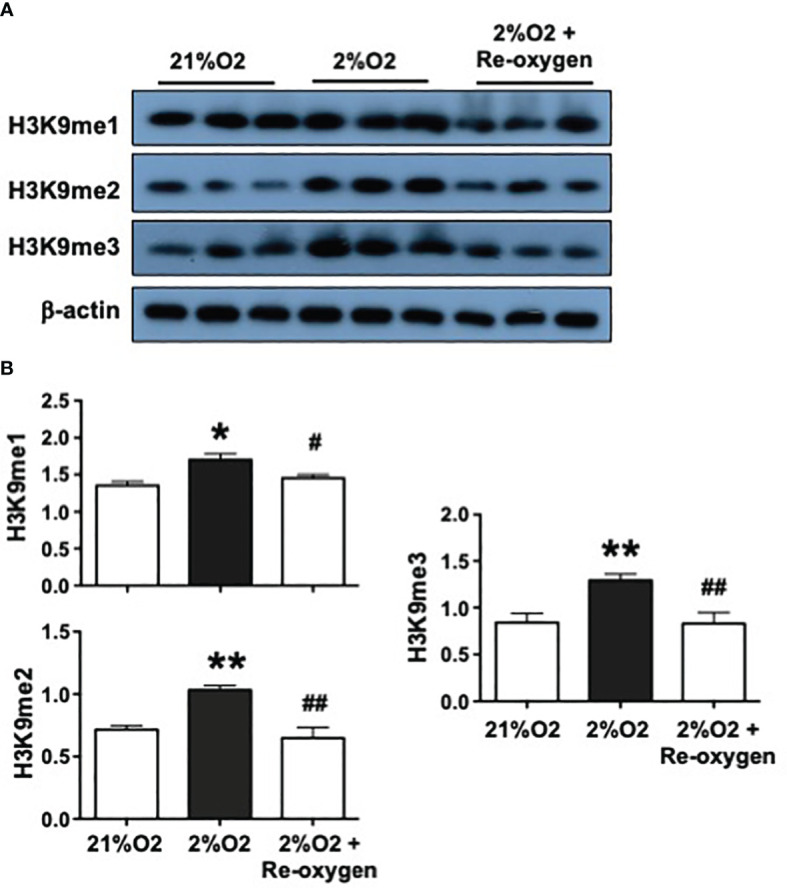
Hypoxia upregulates H3K9 methylation in placental trophoblasts. To determine if H3K9 methylation status is regulated by oxygen environment, H3K9me1, H3K9me2, H3K9me3 expression were examined in trophoblasts cultured under hypoxia and reoxygenation conditions. Hypoxic condition: HTR-8/SVneo cells were cultured 2%O2 for 24 hours; Hypoxic + Re-oxygen condition: HTR-8/SVneo cells were cultured under 2%O2 for 24 hours and then incubated under 21%O2 for 6 hours. Cells cultured under 21%O2 served as control. **(A)** Expression of H3K9me1, H3K9me2, and H3K9me3 were significantly upregulated in cells cultured under 2%O2 but returned to the control levels after cells were reoxygenated at 21%O2 condition. **(B)** The bar graphs show relative expression for H3K9me1, H3K9me2, H3K9me3 after normalized by β-actin expression in each sample. Bar graphs shows mean ± SE from 3 independent experiments. *p<0.05 and **p<0.01: Cells cultured under 2% vs. 21%O2; #p<0.05 and ##p<0.01: 2%O2+ re-oxygen vs. 2%O2 alone. These results indicate oxygen environment could regulate methylation status of H3K9 in placental trophoblasts.

To test if methylated H3K9 expression in placental trophoblasts is regulated by oxygen environment, the expression of H3K9me1, H3K9me2, and H3K9me3 were determined in trophoblasts cultured under hypoxia and re-oxygenation conditions. HTR-8/SVneo trophoblasts were used in this experiment. HTR-8/SVneo trophoblasts were first cultured under regular cell culture condition and then the cell culture plates were placed into a portable air chamber and cultured for 24 hours, which was flushed with 2%O2 (2%O2/5%CO2 balanced with N2). For the hypoxic + re-oxygenation culture, after HTR-8/SVneo cells were cultured under 2%O2 condition for 24 hours, the plates were then transferred to a regular cell culture incubator and continued to be cultured for 6 hours. Cells cultured under 21%O2 only were served as control. Interestingly, as shown in **Figure 2A**, the expression of H3K9me1, H3K9me2, and H3K9me3 was significantly upregulated in cells cultured under 2%O2, and then returned to the control levels after cells were re-oxygenated at a regular oxygen cell culture condition. The bar graphs show relative H3K9me1, H3K9me2, and H3K9me3 expression after normalized by β-actin expression in each sample, **Figure 2B**. These results suggest that variations in oxygenation could regulate H3K9 methylation status in placental trophoblasts with hypoxic condition promoting H3K9 methylation.

### Effects of histone methyltransferase inhibition on antioxidant CuZn-SOD and Mn-SOD expression in placental trophoblasts

**Figure 3:**
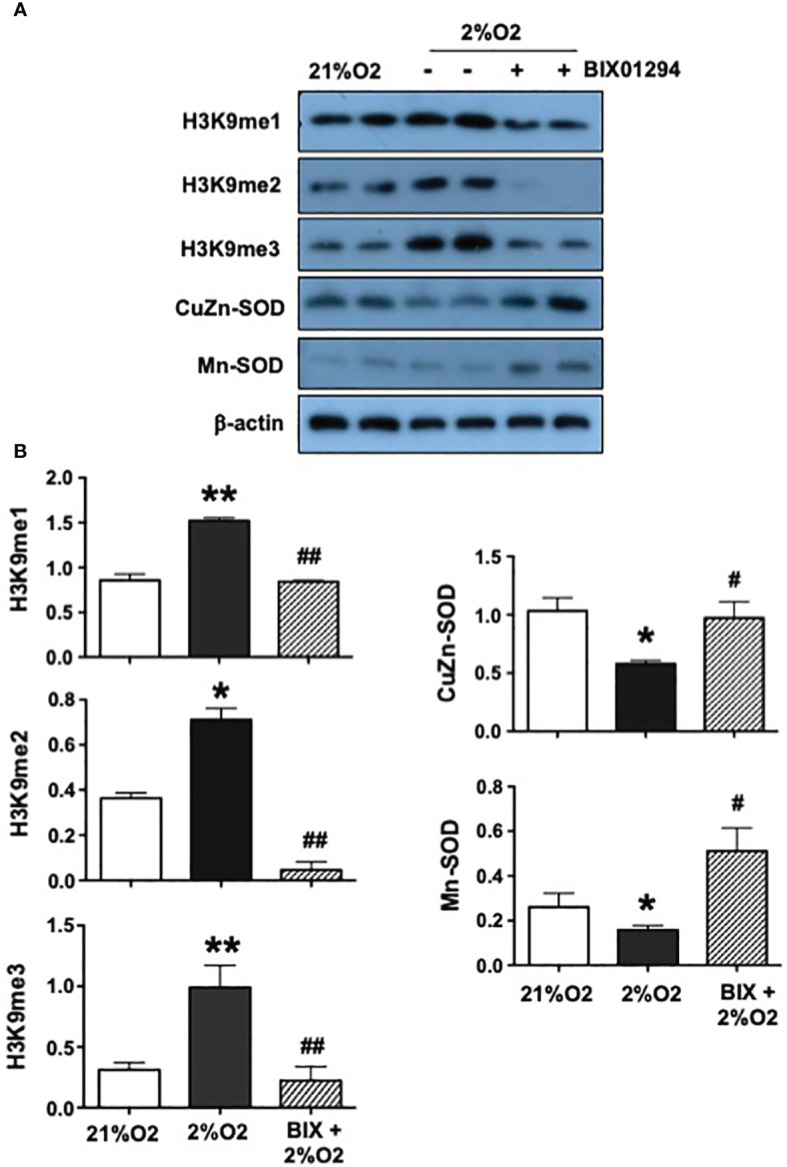
Effects of histone methyltransferase on H3K9me1, H3K9me2, H3K9me3, CuZn-SOD, and Mn-SOD expression in placental trophoblasts. To determine if H3K9 methylation regulates CuZn-SOD and Mn-SOD expression in placental trophoblasts under oxidative stress condition, HTR-8/SVneo cells were cultured under 2%O2 treated with or without BIX01294 (5µM) in culture for 24 hours. BIX01294 is an inhibitor of histone methyltransferase. Expression of H3K9me1, H3K9me2, H3K9me3, CuZn-SOD and Mn-SOD were then evaluated. **(A)** Expression of H3K9me1, H3K9me2, and H3K9me3 were significantly upregulated in cells cultured under 2%O2 alone but no change (H3K9me1 and H3K9me3) or reduced (H3K9me2) in cells treated with BIX01294. In contrast, expression of CuZn-SOD and Mn-SOD were downregulated in cells cultured under 2%O2 alone but increased in cells treated with BIX01294. **(B)** The bar graphs show relative expression for H3K9me1, H3K9me2, H3K9me3, CuZn-SOD, and Mn-SOD after normalized by β-actin expression in each sample, mean ± SE from 5 independent experiments. *p<0.05 and **p<0.01: Cells cultured under 2% vs. 21%O2; #p<0.05 and ##p<0.01: BIX +2%O2 vs. 2%O2. These results suggest that antioxidant CuZn-SOD and Mn-SOD expression are regulated by methylation status of H3K9 in placental trophoblasts.

To determine if downregulation of CuZn-SOD and Mn-SOD expression is associated with hypermethylation of H3K9 expression in preeclamptic trophoblasts, HTR-8/SVneo cells were cultured under 2%O2 in the presence and absence of BIX01294 (5µM) in culture for 24 hours. BIX01294 is an inhibitor of histone methyltransferase. The expression of methylated H3K9 (H3K9me1, H3K9me2, H3K9me3), CuZn-SOD, and Mn-SOD was then determined. As shown in **Figure 3A**, the expression of H3K9me1, H3K9me2, and H3K9me3 was significantly upregulated in cells cultured under 2%O2 alone. However, the level of H3K9me1, H3K9me2, and H3K9me3 expression was either unchanged or reduced in cells treated with BIX01294 when cultured under 2%O2 condition. In contrast, expression of CuZn-SOD and Mn-SOD was downregulated in cells cultured under 2%O2 alone but increased when cells were treated with BIX01294 and cultured under 2%O2 condition. The bar graphs, **Figure 3B**, show relative H3K9me1, H3K9me2, H3K9me3, CuZn-SOD, and Mn-SOD expression after normalized by β-actin expression in each sample. These results showed that inhibition of methyltransferase prevented hypoxia-induced hypermethylation of H3K9 and downregulation of CuZn-SOD and Mn-SOD expression in placental trophoblasts, which further demonstrated that hypoxia promotes histone modification associated with H3K9 methylation and CuZn-SOD and Mn-SOD expression is likely regulated by H3K9 methylation status in placental trophoblasts.

## Discussion

In the present study, we found that methylated histone H3K9 expressions, including H3K9me1, H3K9me2 and H3K9me3, were all significantly upregulated in trophoblasts from preeclamptic pregnancies compared to those from control placentas of normal pregnancies. These findings suggest that hypermethylation of H3K9 in placental trophoblasts occurs in preeclampsia, which support the notion that aberrant histone H3K9 modification noted in preeclamptic placentas. We also found that downregulation of antioxidant superoxide dismutase CuZn-SOD and Mn-SOD expression was associated with upregulation of methylated H3K9 expression in trophoblasts from preeclamptic placentas compared to those from normal control placentas. Superoxide dismutase is the first line of antioxidative defense to eradicate extra superoxide radicals that are generated in biological cells. Downregulation of CuZu-SOD and Mn-SOD expression is a sign of increased oxidative stress and decreased antioxidative activity. These results are important since increased oxidative stress/reduced antioxidant activity is one of the key pathophysiological outcomes in preeclampsia placenta (16–18).

Oxidative stress is a widely occurring phenomenon in biological systems. To test if the hypermethylation status of H3K9 seen in preeclamptic trophoblasts is a consequence of increased oxidative stress, we determined the methylated H3K9 expression in HTR-8/SVneo cells that were cultured under lowered oxygen and re-oxygenation conditions. HTR-8/SVneo is an established trophoblast cell line originally isolated from human first trimester placenta (19) and transformed with the gene encoding for simian virus to large T antigen. HTR-8/SVneo cells exhibit a variety of marker characteristics of extra-villous invasive trophoblast cells and have been widely employed by the research community to study trophoblast function in the past three decades. Strikingly, we found that expression of the three H3K9 methylated forms, H3K9me1, H3K9me2 and H3K9me3, were all significantly upregulated when cells were cultured under lowered oxygen condition. Most interestingly, expression of H3K9me1, H3K9me2, and H3K9me3 were all returned to the relative control levels when cells were re-oxygenated after lowered oxygen culture. These results clearly show that hypoxic condition promotes methylation of histone H3K9, and hypoxia-induced hypermethylation of H3K9 is a dynamic and likely a reversible process in placental trophoblasts.

We previously reported that expression of H3K9me2 and H3K9me3, but not H3K9me1, were upregulated in umbilical cord vein fetal endothelial cells (HUVECs) from preeclamptic placentas (9). In the present study, we found all three methylated forms of H3K9 (H3K9me1, H3K9me2 and H3K9me3) were upregulated in trophoblasts from preeclamptic placenta. Umbilical cord vein transfers oxygenated blood from the placenta to the fetus, while placental trophoblasts contact to maternal blood components that circulate between maternal vascular system and placental intervillous space. It is interesting to note that the expression of H3K9me2 and H3K9me3 was upregulated in both placental trophoblasts and fetal vessel endothelium in preeclampsia, which provide plausible evidence that a relatively hypoxic condition is present not only in the placental intervillous space, but may also be in the fetal circulation, in pregnant women complicated with preeclampsia compared to those uncomplicated control pregnancies.

It is known that methylation of H3K9 has been implicated in heterochromatin formation and gene silencing. High-resolution mapping of histone modification patterns revealed that di- and tri-methylations of Lys-9 (H3K9me2 and H3K9me3) are enriched in the transcriptional start sites of silenced genes, whereas H3K9me1 is present in the promoter regions of active genes (20). Although, the reason for the difference in H3K9me1 expression between trophoblasts and HUVECs is unclear in preeclampsia, based on our data, it is likely that either trophoblasts may experience relatively lower hypoxic conditions than HUVECs or HUVECs may have better ability to compensate for the hypoxic conditions in preeclampsia. The findings of downregulation of CuZn-SOD and Mn-SOD associated with upregulation of methylated H3K9 imply that reduced CuZn-SOD and Mn-SOD expression seen in trophoblasts from preeclamptic placentas could be due to transcriptional repression effect of hypermethylation of H3K9 (H3K9me2 and H3K9me3).

Histone H3K9 methylation is regulated by methyltransferases (HMTs). Several HMTs are present in mammals, including Suppressor of variegation 3–9 homologue 1 (SUV39H1), SUV39H2, SET domain bifurcated 1 (SETDB1), SETDB2, G9A and G9A-like protein (GLP). Previous studies showed that H3K9 methylation could regulate transcriptional repression in placental trophoblast stem cells which is regulated by methyltransferases. For example, Wang et al. found that knockout of methyltransferase SUV39H2 in mouse trophoblast stem cells induced significant changes in cell proliferation and differentiation associated with H3K9 methylation (21). Although our study does not specifically assess transcriptional regulation of CuZn-SOD and Mn-SOD genes, using BIX01294, an inhibitor of histone methyltransferase G9A, we tested the putative role of specific H3K9 methyltransferases on CuZn-SOD and Mn-SOD expression in placental trophoblasts. As we expected, upregulation of H3K9me1, H3K9me2, and H3K9me3 expression was inhibited in trophoblasts with BIX01294 present in culture when cells were cultured under hypoxic condition. Most significantly, we found that hypoxia-induced downregulation of CuZn-SOD and Mn-SOD expression was prevented or even upregulated in trophoblasts when BIX01294 was present in cultures under the lowered oxygen condition. These results further suggest that downregulation of CuZn-SOD and Mn-SOD expression is likely the consequence of hypermethylation of H3K9 in placental trophoblasts in preeclampsia, and inhibition of H3K9 methyltransferases eliminated the repression effect of methylated H3K9 on CuZn-SOD and Mn-SOD genes.

In our hypoxia experiment, cells were cultured under 2%O2, and cells cultured at 21%O2 served as control. Although the condition may not be exactly the oxygen tension in *in vivo* organs and 21%O2 may be considered hyperoxia condition, many investigators used 1 or 2%O2 as lowered oxygen or hypoxic condition for *in vitro* oxidative stress studies. As we know that oxygen transport is different *in vivo* from *in vitro* cell culture conditions. *In vivo*, oxygen is transported to tissues and organs by red blood cell hemoglobin (Hb), while *in vitro* cells are cultured in artificial media that do not contain oxygen carriers. It is known that during pregnancy, oxygen levels are different between the systemic vasculature and placental circulation (22). Several investigators had conducted studies to evaluate levels of oxygen tension in *in vitro* studies. For example, Mackova et al. did a study to test oxygen tension in varied oxygen-buffered medium (23). They found that oxygen tension was 14-16 mmHg in a 2%O2 incubator; 34-42 mmHg in a 5%O2 incubator; and 138-142 mmHg in an air incubator (air/5%CO2 = 21%O_2_). These investigators concluded that these measurements approximate Henry’s Law theoretical value of 15 mmHg, 38 mmHg, and 140 mmHg. Kay et al. conducted a study to test glucose metabolism and hormone release by placental villous tissues in 0%O2, 20%O2, and 95%O2 conditions in an *in vitro* perfusing system (24) and found that glucose consumption was lowest in tissues with 0%O2, and both lactate and lactate dehydrogenase release were lowest in tissues with 95%O2, while tissues with 20%O2 provided an optimal support for hormonal release and functional performance (24). Their data suggest that an approximate 20%O2 environment may be a favorable condition to study trophoblast function *in vitro*. A study performed by Depoix et al. also revealed that the O2 percentage of the air does not negatively affect *in vitro* placental cytotrophoblast differentiation (25). They assessed effects of different oxygen levels on placental trophoblast function and found that there were no differences in cell viability and differentiation between cells cultured at 21%O2 and 8%O2, but fusion appeared to be enhanced under 21%O2 compared to 8%O2 (25). We also published several studies using 2%O2 as a hypoxic condition to study hypoxia induced placental trophoblast function related to preeclampsia (26–28). Moreover, the study reported by Zhou et al. also demonstrated that cells cultured under 2%O2 was relatively compatible to the oxygen environment in preeclamptic placentas (22). Therefore, we believe that using 21%O2 as a relatively normoxic condition and 2%O2 as a hypoxic condition to study oxidative stress mediated trophoblast function are appropriate.

As mentioned early, hypoxia/increased oxidative stress is a key pathophysiological characteristic in placental trophoblasts in preeclampsia. Several studies demonstrated that primary human trophoblasts could mimic preeclamptic phenotype when cells were cultured under hypoxic condition(s), such as increased sFlt-1 production, increased inflammatory cytokine production, upregulation of NRF2 expression, and down-regulation of PLGF expression, etc. (28–30). Hypoxia could also induce trophoblast endoplasmic reticulum (ER) stress as demonstrated by increased GRP78 and GRP94 expression (markers of ER stress) (30) and mitochondria stress revealed by downregulation of Mn-SOD expression in the present study.

There are some limitations in our study. All placentas in the preeclamptic group and 6 out of 7 in the control group are from Africa American women. This disproportionate population represents the demographic and ethnic disparities in the Shreveport community. In addition, in the preeclamptic group, 6 out of 7 placentas are delivered by caesarean section and 6 out of 7 placentas are from female newborns. Although it is not known, it is unlikely that delivery mode and newborn gender had any effect on hypermethylation of histone H3K9 expression seen in trophoblasts from preeclamptic placentas. It is also noticed that gestational age at delivery and the mean placental weight are less in the preeclamptic group than in the control group. Whether gestational age and placental size impact on aberrant epigenetic regulation on placental trophoblasts need further investigation.

## Conclusion

To the best of our knowledge, this is the first study to demonstrate the upregulation of all three forms of methylated histone H3K9 in placental trophoblasts from preeclamptic compared to cells from normal pregnancies. In addition, our study also demonstrated that hypoxia promotes H3K9 methylation and hypermethylation of H3K9 expression in trophoblasts can be abolished when hypoxia condition is removed. These results highlight that H3K9 methylation is a dynamic process and may be potentially reversible in placental trophoblasts. Our study further revealed that the antioxidant superoxide dismutase CuZn-SOD and Mn-SOD are likely regulated by H3K9 methylation since inhibition of H3K9 methyltransferase not only prevents hypoxia-induced upregulation of methylated H3K9, but also blocks hypoxia-induced downregulation of CuZn-SOD and Mn-SOD, expression in placental trophoblasts. These results provide further evidence that aberrant histone modification may have significant impact on placental trophoblasts in pregnancy disorders, such as in preeclampsia. Further study may warrant a focus on the downstream effects of hypermethylation of H3K9 mediated placental trophoblast dysfunction in preeclampsia.

## Data availability statement

The original contributions presented in the study are included in the article/supplementary material. Further inquiries can be directed to the corresponding author.

## Ethics statement

Collection of placentas after delivery from normal and preeclamptic pregnancies was approved by Institutional Review Board (IRB) at LSU Health Sciences Center – Shreveport (LSUHSC-S). The IRB ID number is H97-609 entitled “Altered trophoblast and endothelial function in preeclampsia”.

## Author contributions

YG: Writing – original draft, Methodology, Data curation, Conceptualization. DC: Writing – review & editing, Resources. DL: Writing – review & editing, Conceptualization. DZ: Writing – review & editing, Conceptualization. YW: Writing – review & editing, Writing – original draft, Validation, Methodology, Formal analysis, Data curation, Conceptualization.
